# Cancer incidence and mortality in Manizales 2003-2007

**Published:** 2012-12-30

**Authors:** Guillermo López Guarnizo, Nelson Arias Ortiz, Walter Arboleda Ruiz

**Affiliations:** a Cancer Registy of Manizales. Departament of Maternal and Child Health, Universidad de Caldas, Manizales E-mail Address: guillermo.lopez_g@ucaldas.edu.co.; b Universidad de Caldas,Departament of Públic Health, Universidad de Chile,Departament of Públic Health, Manizales, Departament of Públic Health, Universidad de Caldas, Manizales Colombia, Departament of Públic Health, Universidad de Chile. E-mail Address: nelson.arias@ucaldas.edu.co; c Departament of Maternal and Child Health, Universidad de Caldas, Manizales Colombia. E-mail Address: walter.arboleda@ucaldas.edu.co

**Keywords:** Neoplasm, incidence, mortality, population surveillance, population registries

## Abstract

**Objective:**

To describe cancer incidence and mortality in Manizales during the 2003-2007 period from population-based information.

**Methods:**

The information was obtained from the Manizales Cancer Registry and DANE. We analyzed new cases and cancer deaths of individuals residing in Manizales from 1 January 2003 to 31 December 2007. Cases reported correspond to primary invasive malignant tumors, in all locations, except basal cell carcinoma of the skin. We checked the internal consistency of the data and applied quality indicators suggested by the IARC. The population at risk was obtained from population projections (1985 -- 2020, DANE). Specific rates were estimated by gender and age (18 quinquennial groups), and standardized to the world population directly referenced.

**Results:**

There were 3416 new cases and 1895 deaths from cancer. The age- standardized incidence rate (ASR) per 100,000 people-years for all primary locations (except skin) was 162.4 in women and 166.2 in men. Cancer accounted for 19.8% of mortality in Manizales with ASR per 100,000 people-years of 92.1 in men and 83.6 in women.

**Conclusions:**

The risk of developing cancer or dying from cancer in Manizales is intermediate and similar to national estimates. The information generated by the PCR-M meets international quality standards, so it is necessary to ensure sustainability and improvement.

## Abstract (es)

**Objetivo:**

Describir la incidencia y mortalidad por cáncer en Manizales en el quinquenio 2003- 2007 a partir de información de base poblacional.

**Metodos:**

La información fue obtenida del Registro poblacional de cáncer de Manizales y del DANE. Se analizaron los casos nuevos y las muertes por cáncer ocurridas en personas residentes en Manizales entre el 1º de enero de 2003 y el 31º de diciembre de 2007. Los casos registrados corresponden a tumores malignos invasivos primarios, en todas las localizaciones, incluidos carcinomas escamocelulares de piel y se excluyeron carcinomas basocelulares de la piel, aunque los escamocelulares no se tienen en cuenta en los análisis por su baja mortalidad. Se verificó la consistencia interna de los datos y se aplicaron indicadores de calidad sugeridos por la IARC. La población en riesgo se obtuvo de las proyecciones de población 1985-2020 del DANE. Se estimaron tasas específicas por sexo y edad (18 grupos quinquenales), y se estandarizaron directamente con la población mundial de referencia.

**Resultados:**

Se registraron 3.416 casos nuevos y 1.895 muertes por cáncer. La Tasa de Incidencia Ajustada por Edad (TIAE) por 100.000 personas-año para todas las localizaciones primarias (excepto piel) fue de 162,4 en mujeres y 166,2 en hombres. El cáncer representó el 19,8% de la mortalidad en Manizales con TMAE por 100.000 personas-año de 92,1 en hombres y 83,6 en mujeres.

**Conclusiones:**

El riesgo de desarrollar cáncer o morir por esta causa en Manizales es intermedio y similar a las estimaciones nacionales. El RPCa-M cumple con los estándares internacionales de calidad, es decir, es preciso garantizar su sostenibilidad y mejoramiento.

## Introduction

Cancer is a worldwide problem that generates great burden of disease, especially for developing countries^1^ ^,^ ^2^. According to the GLOBOCAN 2008^3^ report, 56% of the incidence cases and 63% of the deaths registered occurred in less developed regions of the world. In Colombia, cancer was the third cause of death for the 2000-2006 period, registering 203,907 deaths due to cancer: 100,126 in men and 103,781 in women. The age-standardized national mortality rate (ASMR) for cancer in all locations was 83.0 per 100,000 men and 75.6 per 100,000 women. Some 58.8% of deaths due to cancer in men are represented by stomach, lung, prostate, colon and rectum tumors and leukemia; cervical tumors, as well as stomach, breast, lung, and colon and rectum tumors represented 52% of the deaths due to cancer in women^4^.

In Colombia, the age-standardized Incidence Rate (ASIR) for all locations (except for skin tumors different from melanoma) for the 2000-2006 period was 186.6 and 196.9 per 100,000 people-year for men and women, respectively, according to estimates from the National Cancer Institute (NCI). The departments of Risaralda, Caldas, Antioquia, Valle del Cauca, and Quindío remarkably show the highest incidence rates in the country^5^.

### Population Cancer Registry of Manizales (PCR-M)

The registry is a program sponsored by several state institutions (Universidad de Caldas, National Cancer Institute (NCI), Health Secretary of Manizales, and Direction Territorial de Salud de Caldas), which has as its mission to generate and disseminate reliable information on the cancer incidence and mortality due to cancer within the jurisdiction of the Municipality of Manizales, complying with quality parameters established by International Association of Cancer Registries (IACR), contributing to the consolidation of the National Cancer Information System in Colombia; this system is directed by the NCI, entity under the Ministry for Social Protection. Currently, the PCR-M is an active member of the IACR - organism under the International Agency for Cancer Research (IACR) and it is part of the Colombian Population Cancer Registry Network (Red de registros poblacionales de Cancer de Colombia) along with registries from Cali, Pasto, Bucaramanga, and Barranquilla^6^.

The PCR-M is a population-based cancer registry, which gathers data on malignant neoplasms in all locations and from all age groups. The search for cases is active, that is, PCR-M personnel periodically visit information sources, which include public and private hospitals and clinics, histopathology and hematology laboratories, Radiology and nuclear medicine units clinical laboratories, and centers specialized on oncology care in the city. Likewise, data are consulted on deaths from National Administrative Department on Statistics (DANE, for the name in Spanish). The PCR-M does not use reports of autopsies as routine information source. Ever since it began activities in 2002, and until 2006, the PCR-M conducted active searches of cases residing in Manizales and in 10 other municipalities in the Department of Caldas, but as of 2007 it decided to only gather information corresponding to Manizales, for the purpose of completeness and to optimize the scarce resources assigned to the program.

The aim of this work was to describe the incidence and mortality due to cancer in Manizales for the 2003-2007 period based on data gathered by the PCR-M and data on deaths due to cancer obtained from DANE for said period.

## Materials and Methods

### Type of study. Descriptive Population at risk and area of influence.

**Figure 1:**
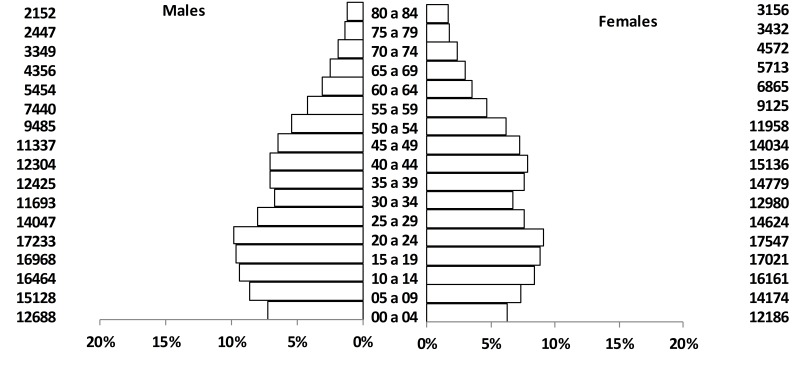
Population structure by age and gender. Manizales, 2005

The PCR-M covers the urban and rural population of Manizales, a municipality located at 2,150 masl on the central range of the Colombian Andean region (coordinates: 05°04'N; 75°31'W), and with an estimated population of 368,433 inhabitants (174,970 men and 193,463 women), according to the last official census from 2005^7^. The municipality's territory covers a total land area of 571.84 km2. Its economy is mainly based on coffee growing and in the tertiary sector (commerce, educational and financial services, among others). Figure 1 shows the population structure by gender and age, according to the most recent census.

### Healthcare infrastructure, screening, and early detection.

By 2007, the Municipality of Manizales had 18 Healthcare Promotion Companies (EPS, for the name in Spanish) in the contributive scheme and four in the subsidized scheme; of the latter, only two are currently in operation. The city has a total of 151 Healthcare Services Provider Institutions (IPS, for the term in Spanish) four public, 138 private, and nine mixed) of which 106 are of low complexity, 41 medium complexity, and four of high complexity. Additionally, it has 635 independent healthcare professionals registered with the health authorities^8^. For over a decade, the municipality has had five histopathology laboratories and five uterine-cervical cytology laboratories. During the study period, five medium and high complexity centers operated specialized in managing cancer patients; currently, four of these are in operation. During the five-year period, 38 information sources were visited with coverage for the urban and rural areas.

As part of the obligatory prevention activities from the Sistema General de Seguridad Social en Salud (General System on Healthcare Social Security), healthcare institutions conduct screening of opportunity for breast cancer and cervical cancer, and early detection activities of prostate cancer. These activities are aimed at the population covered by healthcare insurance^9^.

### Definition of cases and incidence.

The PCR-M began activities in 2001 with a pilot test that extended until late 2002, collecting incident and prevalent cases in the city of Manizales and in 10 municipalities of the Department of Caldas, and evaluating operational and administrative aspects. It began collecting new cases occurring in the urban and rural zones of Manizales as of 2003. The cases registered correspond to all primary invasive malignant tumors, in all locations, including squamous cell carcinomas of the skin and excluding basal cell carcinomas. As primary cancer, we understand that originating from a site or tissue that does not correspond to extension, or recurrence, or metastasis of another primary tumor. Basal cell carcinomas of the skin are not included, nor tumors in situ, or tumors of uncertain behavior defined by the International Classification of Diseases for Oncology - 3^rd^ edition (ICD-O 3)^10^. The unit of registry is the tumor and not the patient, which is why, for multiple primary tumors more than one registry may correspond to one patient. Multiple primary neoplasia were defined, classified, and coded by following international guidelines for 2004^11^.

The date of incidence was defined by following the recommendations from the European Network of Cancer Registries (ENCR), in order of priority, thus^12^: 1. Date of the first histological or cytological confirmation, in the following order: a. Date when the specimen was captured (biopsy) b. Date of reception by the pathologist c. Date of pathology report 2. Date of hospital admission. 3. Date of the first outpatient consultation. 4. Date of diagnosis, except for 1, 2, or 3. 5. Date of death (when the death certificate is the only source of information)

### Classification and coding of cases.

The PCR-M gathers information according to IARC regulations on patient variables - identification number, names and family names, gender, date of birth, age at the moment of diagnosis, home address and vital state - and tumor variables - date of incidence, location, histology, degree and behavior. Coding is carried out by a pathologist trained in the application of ICD-O-3^10^ guidelines. The information was entered and handled by using CanReg4 software. The database was reviewed with IARC Cg-R tools^13^ and Registry PlusTM Link Plus 2.0^14^ to identify errors and data duplicity and to verify the internal consistency among the variables according to the evaluation procedures of registries habitually made by IARC^15^. All cases reported as inconsistent were reviewed in the sources of information and pertinent corrections and confirmations were made. Cases obtained via hospital discharge and death certificate entered the verification process through the clinical history search. Cases detected via death certificate, where additional information was not possible, were included in the database as cases identified only via death certificate (ODC).

### Confidentiality of information.

The PCR-M is governed by guidelines on maintenance of confidentiality from the IACR, according to which access to information from the database is only permitted to the records team, always careful not to publish sensitive data (personal data of cases, data from institutions or from medical and paramedical personnel handling patients). The principle of confidentiality guarantees the individual and citizen right to obtain benefit from knowledge that may result from the registry and from the investigation of causes, prevention, and treatment of cancer, always safeguarding sensitive information and understanding that the effective functioning of the registry depends on the supply of confidential information from all information sources required^16^.

### Quality of information.

To evaluate the quality of data from the PCR-M, some indicators were used suggested by the IARC^17^: percentage of cases with microscopic verification (%MV), percentage of cases registered only via death certificate (%ODC), percentage of cases with unknown primary location or poorly defined (%PD), percentage of cases with unknown age (%UA), unknown gender %UG), and unknown diagnostic base %UDB), and the mortality incidence ratio (M:I).

### Estimates of incidence and mortality.

For incidence estimates, all new cases occurring in people residing in Manizales between 01 January 2003 and 31 December 2007 were considered. Data corresponding to 2002 were excluded given that they were considered as pilot test of the activities of the registry. For mortality, all deaths occurring during the same period with ICD-10 codes were included corresponding to malignant neoplasm, according to death certificates from DANE; prior processing of information was carried out for the complete mortality base, eliminating cases residing abroad, cases without information of age, and cases without information of gender (less than 1% of the total number of deaths); finally, poorly specified uterine tumors were redistributed.

As population at risk we used the 1985 - 2020 population projections elaborated by DANE based on census conciliations. Specific rates were estimated by gender and age (18 quinquennial groups), and these were standardized via the direct method by using the world population as reference. Relative frequencies of new cases and deaths were estimated by specific locations. Incidence and mortality data are presented in groups based on the ICD-10 codes for purposes of comparability, following the methodology used by the IARC in Cancer Incidence in Five Continents (CI5)^17^.

## Results

### Global quality indicators.

For all locations, the %MV was 89% in men and 91% in women. The %ODC represented 6.5% of the cases in men and 4.3% in women. Four percent of the cases in women and 4.9% in men were classified as tumors with unknown primary location. No cases were registered without information regarding variables of age, gender, and diagnostic base. Table 1 shows the quality indicators for locations of highest incidence by gender.

**Table 1:**
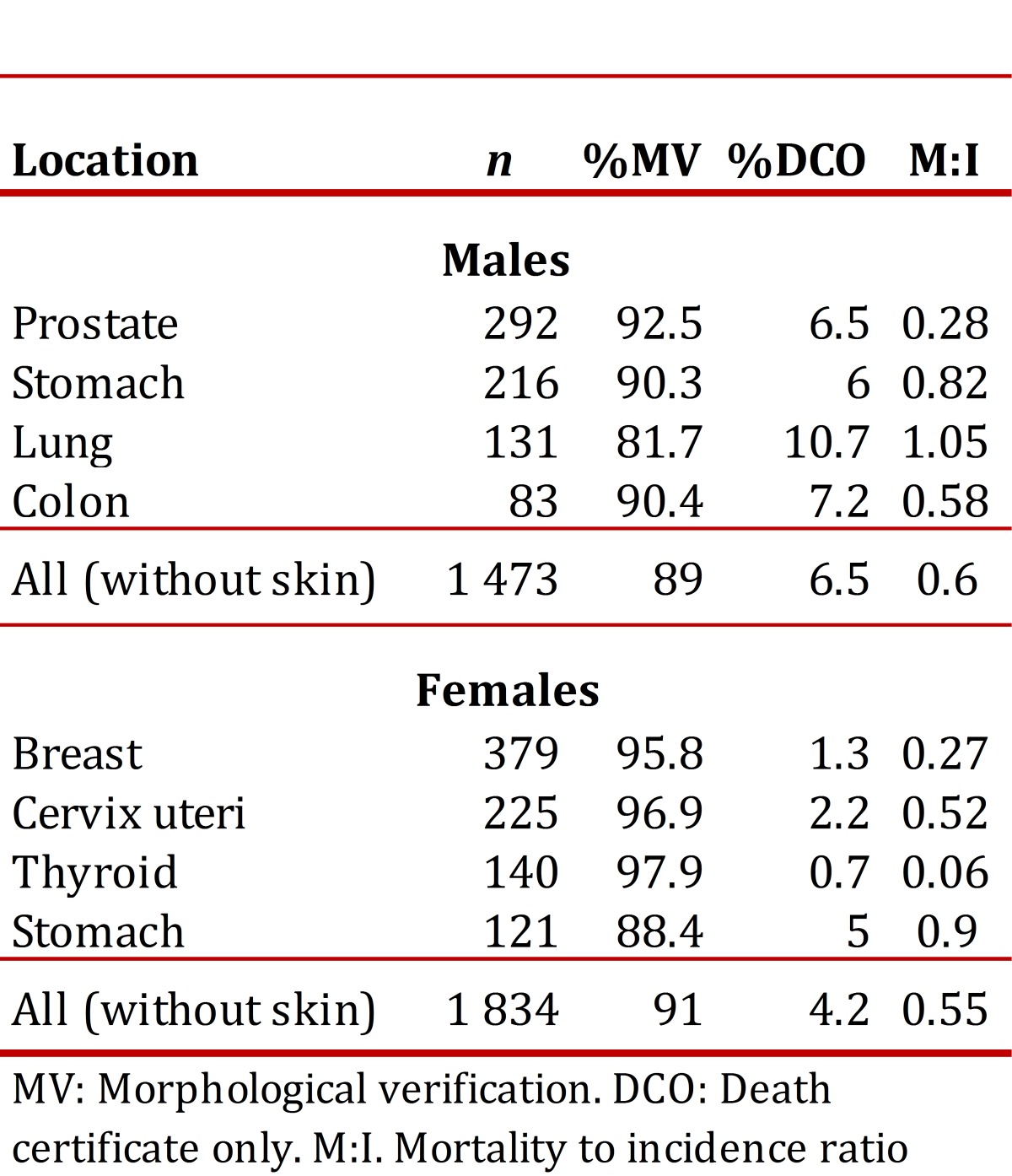
Quality indicators for sities of highest incidence by gender. Manizales Population Registry, 2003-2007.

### Incidence and mortality due to all cancers (all locations)

During the study period, 3416 new cases were registered of which 55.2% were in women. Mean age upon diagnosis was 58 years for women and 61 years for men. Two percent of the cases occurred in pediatric population (younger than 15 years of age). The ASIR per 100,000 people-year for all primary locations (including melanoma and except for the rest of the skin tumors) was 162.4 in women and 166.2 in men. The man/woman incidence rate ratio (IRRm/w) was 1.023.

Of the total number of deaths occurring in Manizales during the study period (n = 9,593), cancer represented 19.8% (n = 1895). In men, proportional mortality due to cancer was 15.8%, while in women cancer caused 25.4% of the total number of deaths. Mortality in men was higher than in women, with ASMR of 92.1 and 83.6 deaths per 100,000 people-year, respectively. Some 56.5% of the male deaths and 51.0% of the female deaths occurred in individuals over 65 years of age; the highest specific rate by age was seen in the group from 75 to 79 years, in both men and women. The mortality rate due to gender (MRGm/w) was 1.1.

### Incidence and mortality due to type of cancer

The five locations of higher incidence in men were: prostate (19.1%), stomach (14.1%), trachea, bronchus and lung (8.6%), colon and rectum (8.6%), and unspecified sites (4.9%), corresponding to 52% of all cancer conditions. In women, the following types of cancer: breast (20.1%), cervix (11.9%), thyroid (7.4%), stomach (6.4%), and trachea, bronchus and lung (5.2%) represent 52.1% of the total number of tumors. Tables 2 and 3 illustrate the incidence and mortality rates according to specific locations by gender.

**Table 2:**
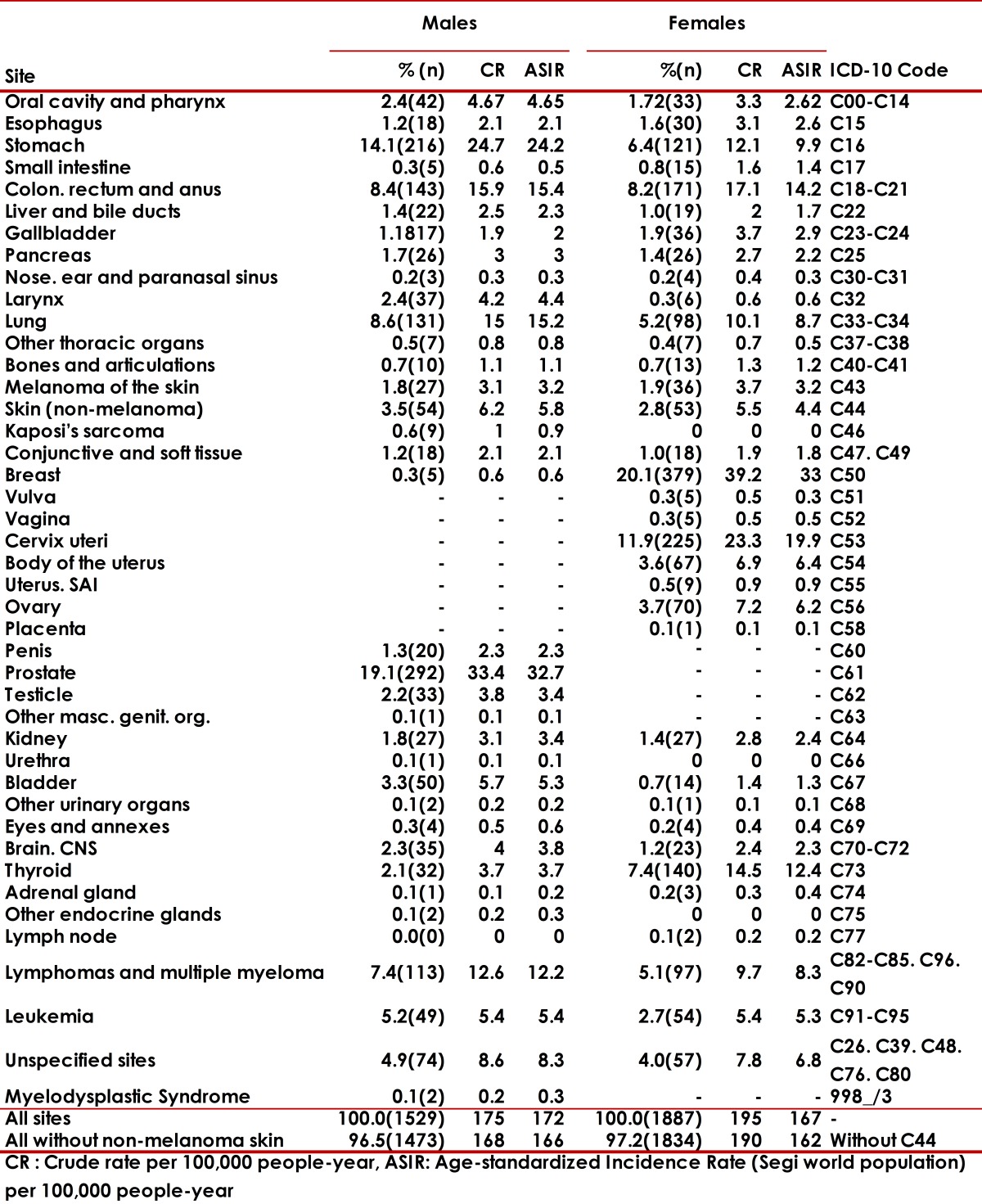
Cancer incidence by site and sex". Manizales, 2003-2007 CR : Crude rate per 100,000 people-year, ASIR: Age-standardized Incidence Rate (Segi world population) per 100,000 people-year

**Table 3.**
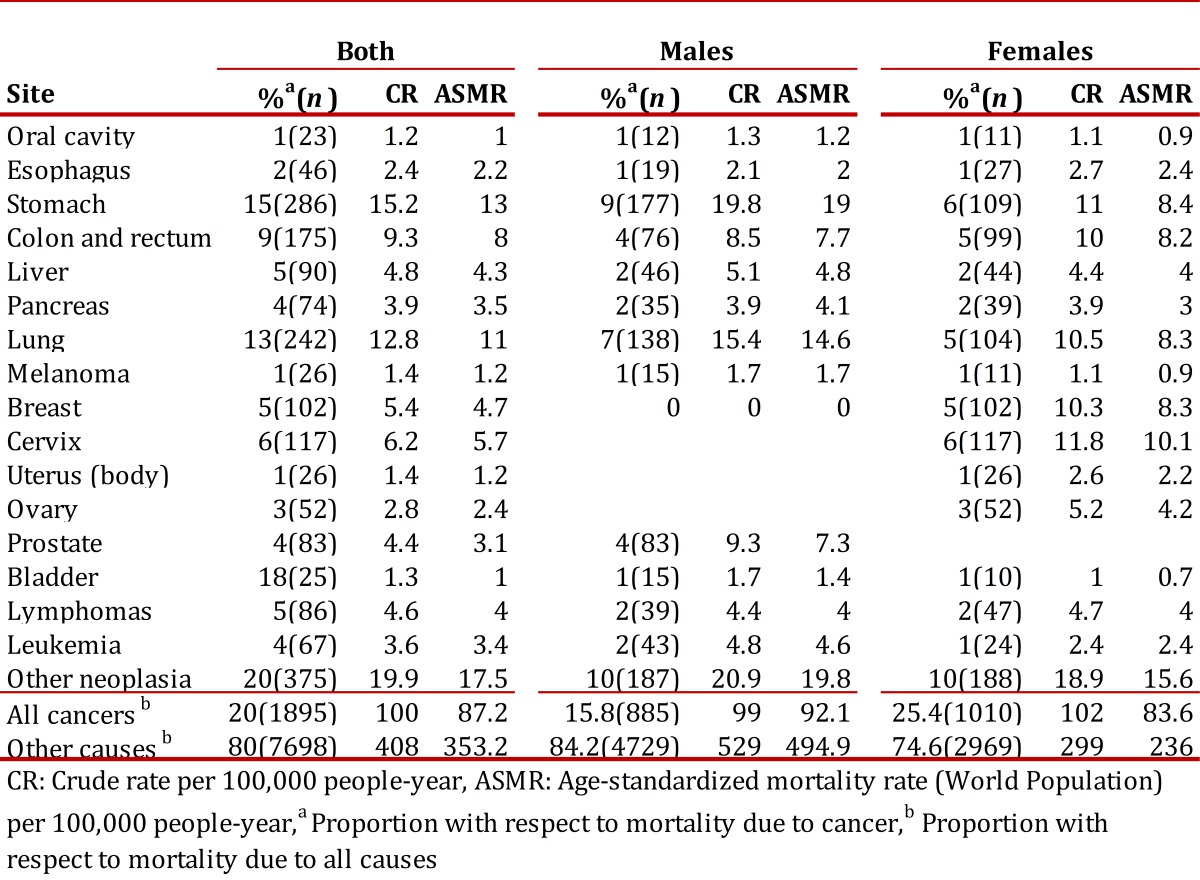
Annual average mortality due to cancer by site and sex. Manizales, 2003-2007 CR: Crude rate per 100,000 people-year, ASMR: Age-standardized mortality rate (Population Mundial) per 100,000 people-year,a Proportion with respect to mortality due to cancer,b Proportion with respect to mortality due to all causes

**Figure 2:**
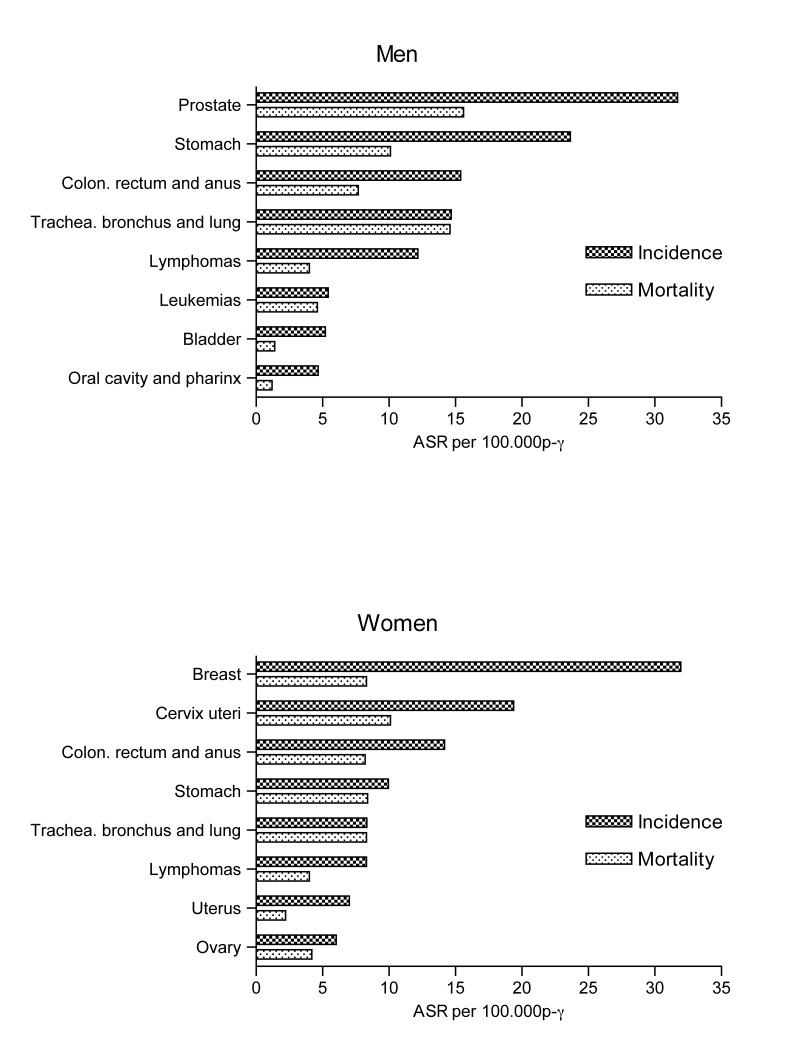
Incidence and mortality due to cancer - First locations by gender (AAR per 100,000 people-year). Manizales, 2003-2007

Stomach (20%), trachea, bronchus and lung (15.6%), prostate (9.4%), colon and rectum (8.6%), and liver (5.2%) tumors account for 58.9% of deaths due to cancer in men. The first five causes of death due to cancer in women were: cervix (11.6%), stomach (10.8%), trachea, bronchia and lung (10.3%), breast (10.1%), and colon and rectum (9.8%); these tumors represent 52.6% of the deaths due to cancer in women. figure 2 illustrates the incidence and mortality rates due to gender for the locations of highest frequency.

### Incidence and mortality according to age.

Leukemia was the most incidental location in girls (0-14 years) with 42.3% of neoplasia in this group, followed by sarcomas with 11.5%. In boys, leukemia and lymphomas account for 53.5% of the malignant tumors in this age group. Leukemia is the main cause of death due to cancer both in boys (38.5%) as in girls (22.2%).

In the group 15-49 years, lymphomas (14.3%) occupy the first place of incidence in men, followed by testicular cancer (11.6%) and by gastric cancer (10.1%); in women, the first places were breast (22%), cervix (19.3%), and thyroid (14.2%). The first causes of death due to cancer for this group were stomach (22.7%), leukemia (12.5%), lung (8.6%), and lymphomas and multiple myeloma (8.6%) in men, and cervix (21.3%), breast (16.5%), ovary (7.9%), colon and rectum (7.9%), and stomach (7.9%) in women.

In men from 50 to 64 years of age, the most incidental tumors were stomach (16.3%), prostate (14.7%), and lung (10.2%); in women from this age group the first locations were breast (25%), cervix (11.6%), and colon and rectum (8.9%). The highest proportions of mortality due to cancer in this age group were tumors of the stomach (21%), lung (17.3%), and colon and rectum (10%) in men, and cervix (13.6%), breast (12.3%), and stomach (11.1%) in women.

**Figure 3:**
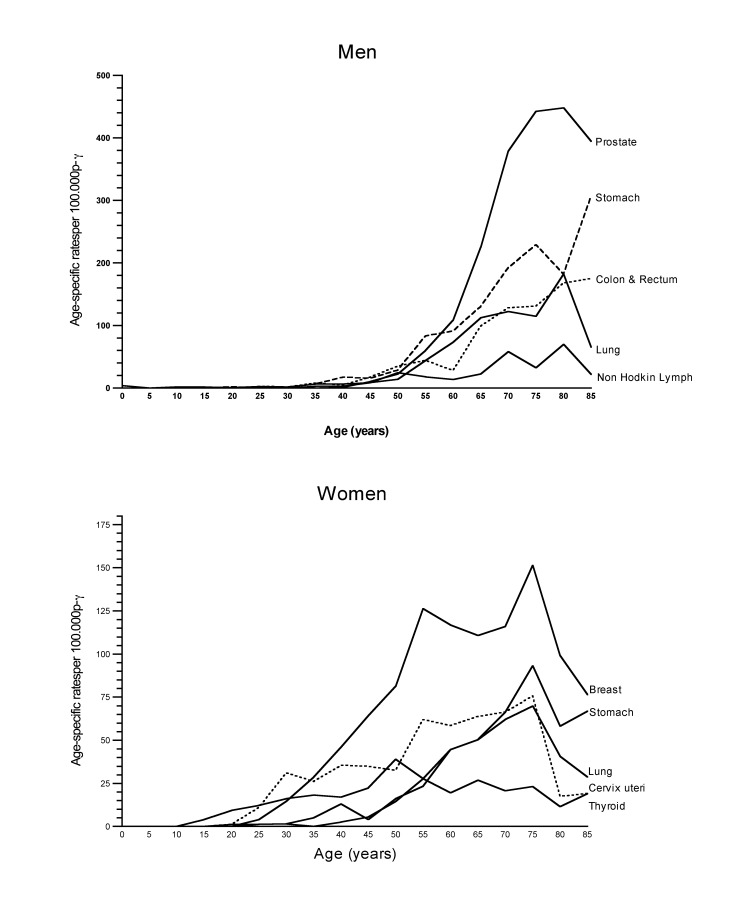
Specific incidence rates by age in women and men, first 5 locations. (Rates per 100,000 p-y). Manizales, 2003-2007

Among men 65 years of age and older, once again the first places of incidence are occupied by tumors of the prostate (28.1%) and stomach (15.1%), followed by colon, rectum and anal cancer (10.3%). Among women in this age group, the highest incidence was noted in tumors of the breast (15.4%), colon, rectum and anus (12.6%), and cervix (7.3%). Mortality was mainly represented by stomach (20.2%), lung (16.8%), and prostate (13.8%) tumors in men, and lung (12.8%), stomach (11.9%), and colon and rectum (10.3%) tumors in women. figure 3 shows the behavior of the cancer conditions with the highest incidence according to age and gender.

## Discussion

The quality criteria of the data indicate that the PCR-M complies with the international requirements defined by the IARC: microscopic verification of at least 80% of the cases and less than 10% of the cases registered via death certificate and from unknown or poorly defined primary site^17^. The M:I ratio > 1 for lung cancer may indicate faults in the completeness of the incidence registry, but may also be due to over registry of lung cancer as the cause of death in death certificates. The information generated by the PCR-M constitutes a valuable contribution to the construction of reliable epidemiological information for the country, which is why the state must guarantee its long-term sustainability and improvement, according to the goals and actions defined in the 6th strategic line of Colombia's Cancer Control Plan (Plan para el Control del Cáncer en Colombia 2012-2020)^18^.

As a set, tumors of gynecological origin (cervix, ovary, uterine body) represent 37% of the female malignant pathology incidence from the period mentioned. Cervical and breast carcinomas represent 32% of the new cases in women, which is corresponded with screening and early detection activities defined in the policies and norms for cancer control in Colombia^19^ ^,^ ^20^. However, studies conducted in the country indicate that for breast cancer, coverage of those activities continues being low^21^.

According to figures reported in GLOBOCAN 2008^3^, breast carcinoma (6655 new cases) occupy the first place in the incidence of cancer in women in Colombia, and the estimated risk of developing this disease is considered intermediate for the country. Mortality continues on the increase in most Latin American countries, as reflected mortality rates in Colombia, Brazil, Mexico, Costa Rica, and Venezuela^22^. In Manizales, risks of developing breast cancer and dying due to this cause are similar to estimates for Colombia^5^.

The incidence of breast cancer in Manizales during the 2003-2007 period (33 per 100,000 people-year) is lower than that reported by Cali's Population Cancer Registry for the same period (48 per 100,000 p-y), but higher than the figures reported by Valdivia (30.9), Quito (30.7), and Uganda (23.4) for the 1998-2002 period; the Bucarmanga registry reported an ASIR of 37.3 per 100,000 p-y for the 2000-2004 period^17^.

The ASIR for cervical cancer in Manizales (19.9 per 100,000 p-y) is below that of the national rate and lower than that reported in Cali (27.9), Valdivia (22.2), Costa Rica (28.9), Brasilia (37.7), and Trujillo (43.9) for 1998-2002; found at the level of cities like Quito (20), Nueva Delhi (19.5), and Bucaramanga (19.9). Nevertheless, we are still far from reaching an incidence similar to that of the United States, Canada, or European countries, whose registries report incidences around 10 per 100.000 p-y, or the incidence of 4 per 100,000 p-y registered by countries like Finland^17^.

In Manizales, only 1% of the malignant tumors of the cervix occurred in women younger than 24 years of age, which supports the recommendation from Colombia's NCI to start screening through uterine cervical cytology as of 21 years of age, and not subject younger women to over treatment because of false positive results^20^. Decreased mortality due to cervical cancer has been attributed in our country to increased coverage of uterine cervical cytology, which reached 76% in 2005^4^.

According to our results, we must assess the benefit of continuing with cytology screening of cervical cancer in women over 69 years of age in Manizales, given that as of 55 years of age, the incidence of cervix carcinoma increases for the whole population, with high rates in age groups that are above the upper age limit defined by the technical norms^20^ ^,^ ^23^

The ASIR for stomach cancer in men in Manizales (24.2 per 100,000 p-y) is lower than that reported by Trujillo (25.2), Cali (27.3), Brasilia (29), and Costa Rica (33.2), and much lower than that reported in Hiroshima (80.3) and Valdivia (43.1), with the last being the highest reported in countries in the Andean range^17^.

In contrast to the marked worldwide mortality decrease^24^, gastric cancer persists as the first cause of death due to cancer in Colombia during the last 30 years, representing 15% of all deaths due to cancer in the country. In our municipality, mortality due to gastric cancer was higher for men and slightly lower for women, compared to national rates. Given that Manizales is in a zone of high incidence and mortality, it is recommended to conduct complementary studies to elucidate the interactions among genetic, environmental, and infectious factors in the causality of this disease.

Regarding lung cancer in men, Manizales presents a lower ASIR (15.2 per 100,000 p-y) than that of Cali (21.5) and Bahia Blanca - Argentina (45.5), but above that reported by Costa Rica (11.1) and Quito (7.9) for the prior five-year period^17^. This condition may be associated to high consumption of tobacco found in the region during the National Study on Consumption of Psychoactive Substances in Colombia^25^.

The ASIR of prostate cancer in the city (32.7 per 100,000 p-y) may be considered low within the international context; it is lower than the incidence for the 1998-2002 period in Cali (63.2), Valdivia (57.6), and Brasilia (101.5), and similar to the rates in Trujillo (27.2) and Quito (39.7). Asia reported the lowest figures for the incidence of this neoplasm, according to registries from Shanghai (6,9) and Seoul (11,7) ^17^. In terms of mortality, in Manizales it was possible to observe lower ASMR than the national figures. The factors related to the incidence and mortality of prostate cancer are known; among these, access to healthcare services and the determination of the prostate-specific antigen, which could lead to increased incidence and to assigning this neoplasm as a cause of death in death certificates.

The incidence of colon cancer (without including rectum and anus) in Manizales is similar to that reported by Cali, Bucaramanga, Quito, and Trujillo, and lower than rates in Northwest Canada, Michigan, and Hiroshima, which are above 40 per 100,000 people-year^17^. Regarding mortality due to this cause, ASMR in Manizales was higher than the national average. According to the NCI, the highest rates were registered in Bogotá, Valle del Cauca, Caldas, and Risaralda^4^. Studies have not been conducted on the prevalence of risk factors (intake of saturated fats, fruits and vegetables, level of physical activity and obesity) in the Manizales population.

Thyroid cancer, in both genders, showed ASIR above those reported by all the population registries from Central and South America, except for the Sao Paulo registry^17^.

## Conclusions

In general, the epidemiological behavior of cancer in the Municipality of Manizales is similar to that found in the country and to that reported during prior years by Cali's Population Cancer Registry. The risk of developing cancer or dying due to this cause is considered intermediate compared to figures reported by other population registries in the region and the world. Incidence due to cancer (all locations, without non-melanoma skin) is lower than the rate estimated for the country, while the mortality is higher than the national estimates. Estimates performed for the 2003-2007 five-year period will serve as a base line for the construction of trends ahead. Knowledge generated by the PCR-M is reliable, according to standards required by the IARC, and constitutes an important contribution to the National System on Cancer Information, given that it permits identifying priorities in planning healthcare services to cancer patients and contributes in accomplishing the objectives, goals, and actions defined by the national policies on the theme. Enhancing the registry will permit optimizing processes, improving the opportunity of information, and expanding the area of influence until encompassing the municipalities that are part of the metropolitan area of Manizales, actions that require guaranteeing its mid- and long-term sustainability.
